# Regulating Acidosis and Relieving Hypoxia by Platelet Membrane-Coated Nanoparticle for Enhancing Tumor Chemotherapy

**DOI:** 10.3389/fbioe.2022.885105

**Published:** 2022-05-12

**Authors:** Xingyu Luo, Jian Cao, Jianming Yu, Dongqing Dai, Wei Jiang, Yahui Feng, Yong Hu

**Affiliations:** ^1^ College of Engineering and Applied Sciences, MOE Key Laboratory of High Performance Polymer Materials & Technology, Nanjing University, Nanjing, China; ^2^ Nanjing Customs District Industrial Products Inspection Center, Nanjing, China

**Keywords:** nanoparticles, chemotherapy, lactate oxidase, platelet membrane, tumor microenvironment

## Abstract

Acidosis and hypoxia of tumor remain a great challenge for cancer therapy. Herein, we developed Hb-LOX-DOX-ZIF8@platelet membrane nanoparticles (H-L-D-Z@PM NPs) to address this problem. Lactate oxidase (LOX) could deplete intratumoral lactate adequately and amplify oxidative stress efficiently. In the meantime, hemoglobin (Hb) was intended to deliver oxygen, relieve hypoxia, and boost the catalytic activity of LOX. The coated PM bestowed active tumor-targeting ability and good biocompatibility to these nanoparticles. Moreover, the encapsulation of zeolitic imidazolate framework-8 (ZIF8) offered the acid response capacity to nanoparticles. With the synergism of chemotherapy drug doxorubicin (DOX), these H-L-D-Z@PM NPs appeared to have excellent antitumor competence. Collectively, this study offered a new strategy for enhancing tumor chemotherapy by regulating acidosis and relieving hypoxia.

## Introduction

Cancer poses a great threat to human health and life (Ferrari, 2005; Zheng et al., 2019). Although a variety of tumor treatment methods have achieved certain clinical effects, there are still plenty of factors that make the treatment performance unsatisfactory (Liu S. et al., 2019; Huo et al., 2019; Xia et al., 2019; Jiang et al., 2020; Xia et al., 2020; Zhang et al., 2020; Jiang et al., 2021). Recently, a large number of studies and clinical evidence show that the acidic environment of tumor tissue is one of the culprits hindering oncotherapy (Binnewies et al., 2018; Zhu et al., 2018; Rohani et al., 2019; Voskuil et al., 2020). The acidosis of tumor is not only an important driving factor for tumor cells and tissues to produce chemotherapy tolerance and immunosuppression (immune escape) but also the key factor for tumor formation and progression promoting the invasion and metastasis of tumor cells (Saha et al., 2016; Rohani et al., 2019). Therefore, regulating the acidic microenvironment of tumor tissues has become one of the hot spots in tumor treatment.

The accumulation of copious amounts of lactic acid is the main cause of the tumor acidic microenvironment (Pillai et al., 2019). In order to obtain the energy required for proliferation, malignant tumor cells consume significant amounts of glucose to meet their energy demands and produce considerable amounts of lactic acid. This biological procedure is called the “Warburg effect” (Warburg et al., 1927). Meanwhile, due to the special physiological structure and rapid proliferation characteristics of tumors, there is low oxygen concentration in the tumor microenvironment. Hypoxia not only promotes solid tumor invasion, metastasis, and recurrence but also exacerbates tumor acidosis (Harris, 2002; Semenza, 2003; Jin et al., 2021). Obviously, the strong correlation between acidosis and hypoxia will result in a compromising therapeutic effect in tumor therapy. Therefore, dissipating lactic acid and alleviating hypoxia simultaneously is the key to regulate the acidic microenvironment of tumors.

As reported in the literature, lactate oxidase (LOX) was used to convert lactic acid to pyruvate and hydrogen peroxide with the participation of oxygen (Gao et al., 2019; Tang et al., 2020; Wang et al., 2022). However, the hypoxic environment of tumor could not offer sufficient oxygen for the reaction. Even worse, this process will exacerbate tumor hypoxia, which undermines the therapeutic effect. Hence, hemoglobin (Hb) is introduced as a carrier to load oxygen, which could relieve hypoxia and increase lactic acid conversion efficiency in cancer therapy (Welter et al., 2016; Otsuka et al., 2018; Yang et al., 2018; Yuan et al., 2021). On this basis, acidosis and hypoxia of the tumor microenvironment could be alleviated; meanwhile, the multidrug resistance (MDR) of the tumor will be diminished.

**FIGURE 1:**
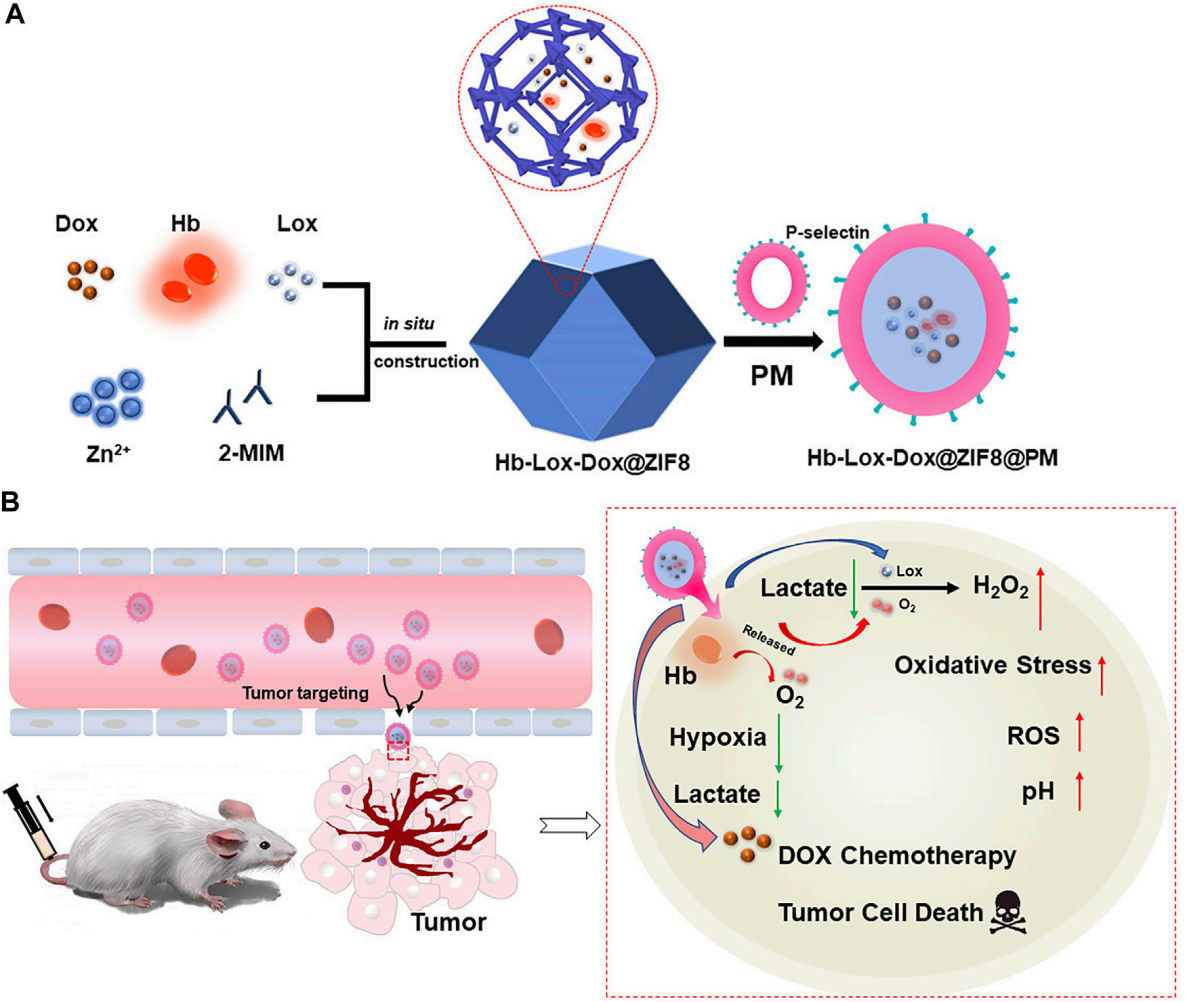
**(A)** Schematic illustration of the main synthesis procedures of H-L-D-Z@PM NPs; **(B)** antitumor mechanism of H-L-D-Z@PM NPs.

In this work, we proposed an innovative nano-drug delivery system loading Hb, DOX, and LOX into the tumor treatment, which could relieve the hypoxia and acidosis in tumor tissue to enhance the chemotherapy effect. Because Hb and LOX are sensitive to the biological environment which could lead to their inactivation, we first encapsulated them in zeolitic imidazolate framework-8 nanoparticles (ZIF8 NPs). Meanwhile, the antitumor drug doxorubicin (DOX) was loaded into ZIF8 to perform chemotherapy (Zhang et al., 2010; Tian et al., 2014; Zheng et al., 2017; Liu et al., 2021; Zhang et al., 2021). Although ZIF8 NPs could passively target tumor tissue through the enhanced permeability and retention (EPR) effect, it still suffered lower targeting efficiency and certain biological toxicity. With respect to this, we coated platelet membrane (PM) on the surface of the composite NPs, which would not only endow the NPs active targeting ability to the tumor but also improve their biocompatibility (Hu et al., 2015; Liu G. et al., 2019; Zhang et al., 2019; Li et al., 2021). There is no doubt that the synergy of active targeting and the EPR effect will significantly increase the accumulation efficiency of NPs in tumor tissue. Thus, a tumor-targeting drug carrier was constructed *via* coating PM on Hb-LOX-DOX-encapsulated ZIF8 to regulate the tumor microenvironment and enhance chemotherapy simultaneously by depleting lactate and relieving hypoxia. When the Hb-LOX-DOX@ZIF8@PM (H-L-D-Z@PM) NPs were injected intravenously into mice, the H-L-D-Z@PM accumulated in the tumor site with high efficiency. After endocytosis, ZIF8 degraded in the slightly acidic environment, releasing LOX and Hb to decompose lactate and alleviate hypoxia. Meanwhile, hydrogen peroxide produced by this process would increase the oxidative stress that leads to the apoptosis of tumor cells. Moreover, the regulated tumor microenvironment will be sensitive to chemotherapy, which could enhance the curative effect of DOX (Figure 1). Taken together, the aforementioned process ultimately regulated the tumor microenvironment and improved the effect of oncotherapy. We expect that our stratagem could provide a promising method for cancer therapy.

## Experimental Section

### Chemicals

Zinc nitrate hexahydrate [Zn (NO_3_)_2_·6H_2_O, AR], 2-methylimidazole (2-MIM, AR), and doxorubicin hydrochloride (DOX, AR) were purchased from J&K Scientific (Beijing, China). Hemoglobin from bovine blood (Hb, AR) was obtained from Shanghai Yuanye Bio-Technology Co., Ltd (China). Lactate oxidase (LOX, ≥90 units/mg solid) and IR-780 iodide (AR) were obtained from HEOWNS Technology Co., Ltd (Tianjin, China). Cell Counting Kit-8 (CCK-8) was supplied by Dojindo Molecular Technologies Inc. The lactic acid assay kit was purchased from Solarbio Life Science Co., Ltd (Beijing, China). Anti-mouse CD62p antibody (ab255822) was purchased from Abcam (Shanghai, China). Pimonidazole hydrochloride and corresponding mouse monoclonal antibody against pimonidazole–protein adducts (FITC-Mab1) were purchased from Hypoxyprobe Inc. (Burlington, MA, United States), and Alexa Fluor 647-labeled rabbit monoclonal HIF-1α antibody were purchased from Abcam (ab208420, Abcam, United Kingdom). All other reagents were used as received and without further purification.

### Cell Lines

Human umbilical vein endothelial cells (HUVECs) and mouse breast cancer cells (4T1) were supplied by the Shanghai Institute of Cell Biology (Shanghai, China). Roswell Park Memorial Institute 1640 (RPMI-1640) medium and phosphate-buffered saline (PBS, pH = 7.4, 6.8, 5.5) were purchased from KeyGen BioTech (Nanjing, China). Fetal bovine serum (FBS) was obtained from Zhejiang Tianhang Biotechnology Co., Ltd. (Hangzhou, China). The hypoxia/oxidative stress detection kit was obtained from Enzo Life Sciences (New York, United States). pHrodo™ Rea AM (Invitrogem™, United States) LysoTracker Deep Red was purchased from Thermo Fisher Scientific Incorporated (America). All cells used in this work were incubated in RPMI-1640 medium supplemented with 10% FBS at 37°C with 5% CO_2_.

### Instrument

The morphology of these nanoparticles was observed by using the FEI TECNAI G2 20 high-resolution transmission electron microscope. Crystalline phases of these materials were measured by XRD (*λ* = 1.54 056 Å, Bruker Co., Ltd., Germany). The UV–vis absorbance spectra of samples were detected by using a UV–vis spectrophotometer (UV3100, Shimadzu, Japan). The ζ-potential and hydrodynamic size distribution of the nanoparticles in water were obtained using a Zetasizer 3000HS analyzer. A JPB-607A dissolved oxygen meter (Rex Electric Chemical, China) was used to measure the O_2_ content. The data in this work were presented as an average of three measurements. The FV3000 confocal laser scanning microscope (Olympus, Japan) was used to acquire confocal fluorescence images. All the tissue sections were prepared by Nanjing KeyGen BioTech and observed by using a fluorescence microscope DMI8 (LEICA, Germany).

### Animal Models

The BALB/c mice (female, 6–8 weeks) were supplied by the Comparative Medicine Centre of Yangzhou University and raised in a specific pathogen-free facility. To set up the breast tumor model, these mice were inoculated subcutaneously with a 4T1 cell line (1 × 10^6^ cells per mouse). All animal experiments were reviewed and approved by the Committee on Animals at Nanjing University, and guidance was provided by the National Institute of Animal Care.

### Acquisition and Purification of Platelet Membrane

We used SD rats of SPF grade to obtain platelets. Briefly, SD rats were intraperitoneally injected with 0.3 ml/kg body weight of 10% chloral hydrate solution. The puncture site was determined by three-line positioning, and blood was collected by the negative pressure of the blood collection tube. Blood samples were centrifuged at 100 g for 20 min at room temperature to separate red and white blood cells. The resulting platelets were centrifuged at 100 g again for 20 min to remove the remaining blood cells. PBS containing 1 mM EDTA and 2 mM prostaglandin E1 (PGE1, Sigma Aldrich) was added to purified blood cells to prevent platelet activation. Platelets were pelleted by centrifugation at 800 *g* for 20 min at room temperature. The supernatant was then discarded, and the platelets were resuspended in PBS containing 1 mM EDTA and mixed with protease inhibitors (Pierce) until used. To extract and purify the platelet membrane, the platelet suspension was frozen at −80°C and then placed at 25°C for five cycles. The platelet suspension was centrifuged at 12,000 g for 20 min at 4°C to collect the platelet membrane. The platelet membrane was stored at −80°C and used for the preparation of different nanoparticle formulations.

### Synthesis of Hb-LOX-DOX-ZIF8@PM (H-L-D-Z@PM) NPs

For the synthesis of H-L-D-Z@PM NPs, 93 mg of Zn (NO_3_)_2_·6H_2_O was first dissolved in 1 ml of DI water, which contained 5 mg of DOX, 10 mg of Hb, and 2 mg of LOX. After stirring at 4°C for 20 min, we added this solution slowly into the solution of 2-methylimidazole (10 ml, 1 M), and the mixture was stirred at 4°C for a further 30 min. Then, the H-L-D-Z NPs were collected by centrifugation and washed three times. In the end, the samples were freeze-dried by a vacuum freeze dryer. As controls, pure ZIF8, Hb@ZIF8 (H-Z), LOX@ZIF8 (L-Z), DOX@ZIF8 (D-Z), and Hb-LOX@ZIF8 (H-L-Z) NPs were synthesized with the same method. Furthermore, in order to coat H-L-D-Z NPs with platelet membrane (PM), 10 mg of H-L-D-Z NPs were dispersed in 1 ml of PBS solution, which contained 8 mg of PM. After sonication and stirring in an ice bath for 10 min, the PM-coated NPs were washed two times with PBS (pH = 7.4, containing 1 mM EDTA and mixed with protease inhibitors). In the end, we obtained the H-L-D-Z@PM NPs by freeze-drying.

### Loading and Releasing O_2_

A JPB-607A dissolved oxygen meter (Rex Electric Chemical, China) was employed to measure the O_2_ content. A measure of 5 mg of Hb, 5 mg of ZIF8 NPs, and 5 mg of H-L-D-Z@PM NPs were first dispersed in 20 ml of PBS, respectively. Then the solution was subjected to pure oxygen saturation for 30 min. The O_2_-loaded NPs were collected by centrifugation. Afterward, the NPs were quickly dispersed in 20 ml of deoxygenated PBS (prepared by boiling under a nitrogen atmosphere) and sealed. The oxygen content in a certain time interval was recorded. The data in this work were presented as an average of three measurements.

### DOX Release Study

The release of DOX was evaluated by measuring the UV–vis absorbance of DOX. First, 5 mg of H-L-D-Z@PM NPs was dispersed in 3 ml of PBS (pH = 5.5, 6.8, and 7.4) to simulate the physiological conditions of normal and tumor tissues, respectively. Then the suspension was poured into a dialysis bag, which was soaked in 10 ml of the same buffer solution, and placed in an oscillator at 37°C. At appropriate time intervals (0.5, 1, 2, 4, 8, 12, 24, 48, and 72 h), 1 ml of soak solution outside the dialysis bag was taken out for UV–vis analysis at a wavelength of 478 nm and measured at predetermined time intervals to estimate the percentage of released DOX. The same volume of fresh buffer solution was added to maintain consistency with the soak solution volume.

### Cytotoxicity Assay

The cytotoxicity of the samples was detected by the CCK-8 test. Briefly, 4T1 and HUVECs were seeded into the 96-well plates (5 × 10^3^ cells per well) and incubated at 37°C overnight. Then, different concentrations of H-L-D-Z or H-L-D-Z@PM NPs dispersed in fresh medium without FBS were employed to replace the culture medium in each well and incubated at 37°C for another 24 h. Finally, 10 µl of CCK-8 was added into each well and further incubated for several hours until the color of the medium became orange. The absorbance of each well at 450 nm was measured with a SPARK Enzyme mark instrument (TECAN Inc., Swiss).

### Intracellular Internalization Analysis

To study the cellular uptake efficiency and track the DOX in cells, H-L-D-Z@PM NPs were incubated with 4T1 cells on a confocal dish (100 µl H-L-D-Z@PM NPs, 2 mg/ml in medium) at 37°C for 2 and 6 h, respectively. After washing with PBS (pH = 7.4) twice, the cells were stained with Hoechst 33342 (1 µl) and LysoTracker Deep Red (1 µl) for 15 min, respectively. Subsequently, the cells were washed with PBS and observed by using a confocal laser scanning microscope (FV3000, Olympus, Japan).

### Oxidative Stress/Hypoxia Detection

The NP-induced oxidative stress/hypoxia generation was detected by using the hypoxia/oxidative stress detection kit (Enzo Life Sciences). For the *in vitro* test, 4T1 cells were seeded in a confocal dish at a density of 1 × 10^5^ per dish and incubated in a complete medium at 37°C. On the next day, the culture medium was removed, and the cells were washed twice with PBS, followed by incubation with fresh media containing different samples (the corresponding ZIF-8 NPs were 150 μg/ml) for 4 h. The hypoxia/oxidative stress detection mixture was added to cell culture media and measured using CLSM imaging (FV3000, Olympus, Japan).

### Intracellular pH Detection

In this study, pHrodo™ Rea AM (Invitrogem™) was employed to evaluate the intracellular pH level. 4T1 cells pre-seeded in confocal dishes (1 × 10^5^ cells per dish) were treated with different samples (the corresponding ZIF-8 NPs were 150 μg/ml) and cultured for another 4 h. After that, the cells were washed with PBS and cultured in the serum-free RMPI-1640 medium containing pHrodo™ Rea AM (1 μM) for 30 min. Then cells were washed thrice with PBS and subjected to CLSM observation.

### Lactate Concentration Analysis of Cell Medium

After culturing at 37°C for 24 h, 4T1 cells pre-seeded in 6-well plates were treated with different samples (the corresponding ZIF-8 NPs were 150 μg/ml) for 24 h. Then cells were lysed with Triton-X-100 lysis buffer and harvested for the ultrasonic process to ensure that the cells were completely broken. The lactate levels were counted by using the lactate assay kit, respectively, and the result was calculated based on the lactate content of untreated cells.

### *In Vivo* Luminescence Imaging

To track the distribution of NPs in the body, *in vivo* luminescence imaging of tumor was performed by using the IVIS Spectrum (Perkin Elmer, America). H-L-D-Z@PM NPs labeled with IR-780 (200 μl, 1 mg ml^−1^ in saline) were intravenously injected into 4T1 tumor-bearing mice. The luminescence images were recorded at various time points. After injection for 72 h, the mice were euthanized, and major organs were harvested and detected by using an *in vivo* imaging instrument.

### Characterization of Platelet Membrane Proteins

The protein expression profiles of platelet membrane and H-L-D-Z@PM were analyzed by Western blot experiments. First, the different suspended samples were centrifuged at 12,000 g for 20 min, and the precipitates were dissolved in pre-cold RIPA buffer on ice for 30 min. The protein solution was collected by centrifuging at 12,000 g for 30 min, and the supernatant was boiled in protein loading buffer for 5 min. For the Western blot experiment, protein gel was transferred into a poly(vinylidene difluoride) membrane and then blocked with 5% BSA for 1 h. The membrane was then incubated with the antibodies of platelet membrane protein markers CD62p for 2 h at room temperature. The membrane was washed three times with TBST solution (Tris-buffered saline with 0.02% Tween 20) and incubated with the IgG-HRP secondary antibody (goat anti-rabbit) for another 1 h. The blot images were visualized by using the Bio-Rad ChemiDoc Touch Imaging System (Bio-Rad, United States).

### *In Vivo* Antitumor Study

The 4T1 tumor-bearing mice were randomly divided into 7 groups (5 mice in each group). When the tumor volume reached about ∼100 mm^3^, all the groups were intravenously injected with an equivalent dose of samples (200 μl, 2 mg ml^−1^ in saline), except the control group (200 µl of PBS), and the same operation was repeated every 3 days. To study the therapeutic effects and the reliability of treatments, the relative tumor volume, the body weight of mice, and the survival of each mouse were recorded every 2 days. The tumor volume was calculated as follows: *V* = W^2^× L/2, where W and L represent the tumor width and length, respectively. The mice were executed after 14 days, and the tumors were collected and photographed. Simultaneously, the major organs (heart, liver, spleen, lungs, and kidney) and tumors of the mice were collected for H and E staining. Furthermore, TUNEL apoptosis staining was employed to assess the tumor cell apoptosis and necrosis. For the labeling of hypoxic cells, pimonidazole was administrated intravenously (60 mg/kg weight) 2 days after the mice received either H-L-D-Z@PM or L-D-Z@PM (dosage as mentioned earlier). Afterward, the mice were sacrificed to harvest the tumor tissues, followed by cryo-section processing and immunofluorescence staining of both HIF-1α and pimonidazole–protein adduct. All these tissue sections were prepared by Nanjing KeyGen BioTech Company and observed by using a fluorescence microscope (DMI8, LEICA, Germany).

### Pathology Analysis

After treating with H-L-D-Z@PM NPs, 4T1 tumor-bearing mice were sacrificed on the 1st, 7th, 15th, and 30th day, respectively, to evaluate the chronic toxicity. The major organs (heart, liver, spleen, lung, and kidney) were collected, sliced, and observed with H and E staining.

### Detection of Lactate in Tumor Tissue

To demonstrate that H-L-D-Z@PM NPs can exhaust lactate in tumor tissue, 14 days posttreatment, the tumors were harvested, triturated, and decolorized, and the lactate in the supernatant was detected by using a lactate assay kit.

### Statistical Analysis

Quantitative data were analyzed by using Student’s *t* test by GraphPad Prism software (version 7.5), and a *p*-value of 0.05 or less was considered to be statistically significant.

## Results and Discussion

**FIGURE 2:**
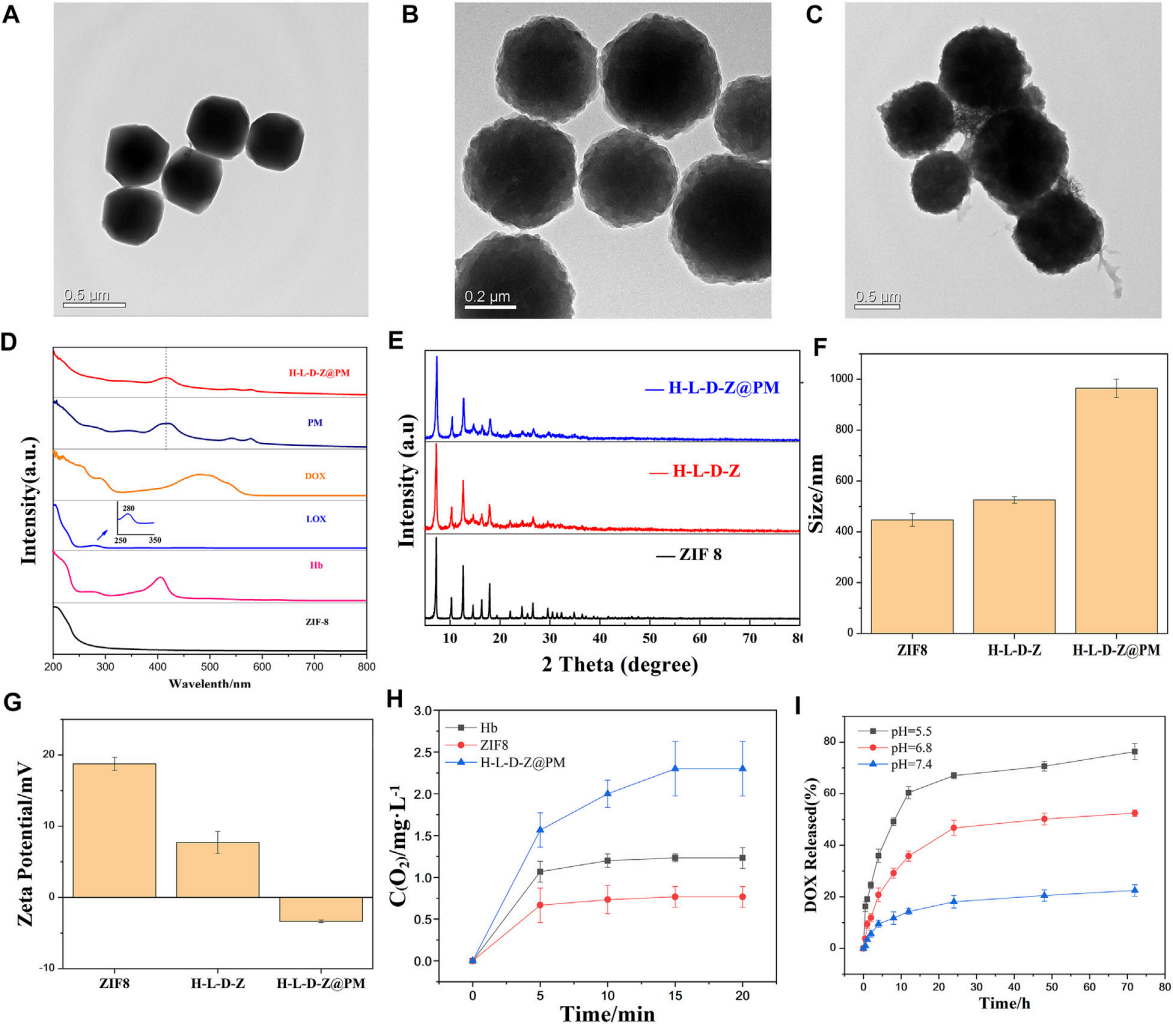
TEM image of **(A)** ZIF8 NPs, **(B)** H-L-D-Z NPs, and **(C)** H-L-D-Z@PM NPs. **(D)** UV–vis absorption spectra of ZIF8, Hb, LOX, DOX, PM, and H-L-D-Z@PM. **(E)** XRD pattern of ZIF8, H-L-D-Z, and H-L-D-Z@PM. **(F)** Hydrodynamic size distribution and **(G)** ζ potentials of ZIF8, H-L-D-Z, and H-L-D-Z@PM. **(H)** Oxygen-carrying and -releasing ability of ZIF8, H-L-D-Z, and H-L-D-Z@PM. **(I)** Effect of pH on the release of DOX from H-L-D-Z@PM.

In this work, H-L-D-Z@PM NPs were synthesized in two steps as shown in Figure 1. First, ZIF8 NPs or H-L-D-Z NPs were synthesized by a simple and efficient one-pot approach as described in the experimental section. Subsequently, H-L-D-Z@PM NPs were fabricated by coating H-L-D-Z with PM. The appearance of these ZIF8 NPs was measured by transmission electron microscopy (TEM). The pristine ZIF8 NPs show a regular polyhedron structure with a smooth surface, whose size is about 447 nm with a positive zeta potential of 18.77 mv due to the presence of 2-methylimidazole (Figures 2A,F). After the encapsulation of Hb, LOX, and DOX, the H-L-D-Z NPs still present a polyhedron structure with a rough surface, and their size increases from 447 to 525 nm concurrently, indicating the loading of LOX, Hb, and DOX (Figures 2B,F). Interestingly, their zeta potential decreased from 18.77 to 7.71 mV after the loading procedure (Figure 2G). When the H-L-D-Z is coated with PM, their hydrodynamic size increases to 964 nm (Figures 2C,F), and their zeta potential decreases from 7.72 mV to −3.35 mV simultaneously (Figure 2G), further confirming the coating of PM. The coating of PM was also verified by the UV-vis spectrum, as illustrated in Figure 2D. After being covered by PM, the UV-vis characteristic absorption peaks of Hb, LOX, and DOX disappear, while the characteristic peak of PM at 393 nm is seen in the H-L-D-Z@PM curve, which indicates that PM coats the surface of H-L-D-Z@PM NPs successfully and compactly. Moreover, we also validated the presence of platelet membrane protein of CD62p on the H-L-D-Z@PM surfaces by Western blot analysis (Supplementary Figure S1), which is the crucial receptor for binding to tumor vascular endothelium. As X-ray diffraction (XRD) analysis (Figure 2E) shows regardless of H-L-D-Z or H-L-D-Z@PM, the diffraction peaks of them are consistent with pure ZIF8, which implies that the coating or encapsulation process would not change their crystal structure. After that, the standard curve revealing the DOX concentration was set up (Supplementary Figure S2) to investigate the loading efficiency and release ability of DOX. H-L-D-Z@PM NPs showed a high loading efficiency of DOX of about 73.63%. We further examined the DOX releasing performance of H-L-D-Z@PM NPs under distinct pH and time, as depicted in Figure 2I. A slow and sustained release of DOX from H-L-D-Z@PM NPs was observed, and less than 20% of the loaded DOX was released even after 72 h at pH 7.4. When these H-L-D-Z@PM NPs were incubated in the acidic medium at pH 6.8, similar to the tumor tissue, a faster release of DOX was obtained, and around 50% of DOX was released after 72 h. When the releasing experiment was conducted in the medium with pH 5.5, which is similar to the lysosome in the tumor tissue, the fastest release of DOX from these H-L-D-Z@PM NPs was obtained. More than 60% of DOX was released within 10 h, and the cumulative release of DOX was over 72% within 72 h. The pH-sensitive release of DOX could be attributable to the dissociation of ZIF8 in the acid environment. As we know that tumor tissue has an acidic environment, these H-L-D-Z@PM NPs are stable in the normal physiological environment and quickly release DOX in the tumor tissue, which could enhance the antitumor effect and avoid damage to normal tissues.

Since H-L-D-Z@PM NPs are also designed to load and release oxygen in tumor tissue, we assessed the performance of loading and release of oxygen by different NPs after the loading of oxygen. Hb, ZIF8, and H-L-D-Z@PM NPs were immersed in the PBS solution to evaluate the release of O_2_. Interestingly, ZIF8 could load a small amount of oxygen and release all the oxygen within 5 min, which could be attributed to the porous structure of ZIF8. For Hb, a higher amount of oxygen was loaded, and most of the oxygen was released within 5 min. After that, the remaining oxygen would be sustained released within 20 min due to the interaction between Hb and oxygen. However, H-L-D-Z@PM NPs showed different release behaviors. The oxygen loading amount in H-L-D-Z@PM NPs was almost three times greater than that in the ZIF8 NPs, and around 65% of oxygen was released within 5 min. After that, these H-L-D-Z@PM NPs showed a sustained release of oxygen (Figure 2H). Collectively, these results indicated that H-L-D-Z@PM NPs could not only release the DOX in the tumor tissue but also deliver oxygen to the tumor tissue to alleviate the hypoxia.

**FIGURE 3:**
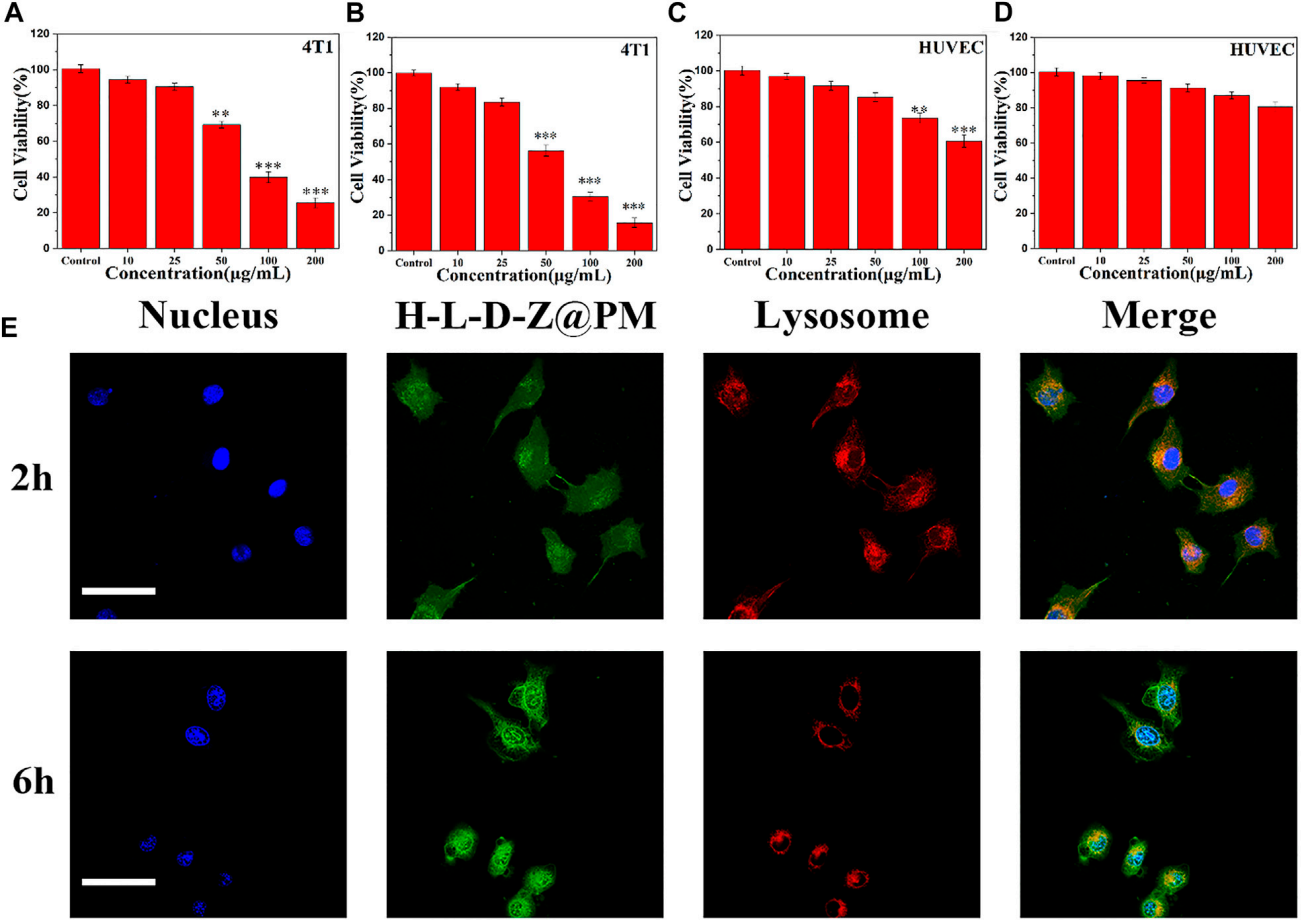
Cell viability of 4T1 cells treated with **(A)** H-L-D-Z NPs and **(B)** H-L-D-Z@PM NPs within 24 h. *In vitro* viability of HUVECs incubated with **(C)** H-L-D-Z NPs and **(D)** H-L-D-Z@PM NPs for 24 h. **(E)** Endocytosis of H-L-D-Z@PM NPs. Scale bars: 10 μm **p < 0.01, ***p < 0.001.

Next, the antitumor ability of H-L-D-Z@PM NPs was evaluated by incubating them with tumor cells. The cytotoxicity of H-L-D-Z and H-L-D-Z@PM NPs was determined by cell counting kit-8 (CCK-8) assay against HUVECs and 4T1 cells. After incubating for 24 h, H-L-D-Z NPs showed low cytotoxicity against HUVECs. When their concentration was lower than 50 μg/ml, more than 80% of the cells were alive. Increasing the concentration would enhance the cytotoxicity, and the cell viability was less than 60% when the concentration reached 200 μg/ml. Covering PM on the surface of H-L-D-Z NPs clearly decreased the cytotoxicity against HUVECs. Even though the concentration of H-L-D-Z@PM NPs was as high as 200 μg/ml, more than 80% of HUVECs were alive (Figures 3C,D). Higher cytotoxicity was observed when these NPs were incubated with 4T1 cells. Less than 70% of 4T1 cells were alive when 50 μg/ml of H-L-D-Z NPs were incubated with 4T1 cells. Compared to H-L-D-Z NPs, the H-L-D-Z@PM NPs revealed a more lethal ability to 4T1 cells (Figures 3A,B), and less 4T1 cells were alive at the same concentration. These results might be attributable to the coating of the PM, which could specifically identify and target tumor cells actively, enhancing the accumulation of H-L-D-Z@PM NPs in tumor cells so that it intensifies the antitumor activity of H-L-D-Z@PM. Consequently, the coating of PM not only augments the antitumor capability of H-L-D-Z@PM but also improves its biocompatibility.

Subsequently, to further investigate the interaction between H-L-D-Z@PM NPs and cells, the cellular uptake of H-L-D-Z@PM by 4T1 cells was investigated by using a confocal laser scanning microscope (Figure 3E). Apparently, the vast majority of H-L-D-Z@PM NPs (green, DOX) were assimilated into 4T1 cells within 2 h and overlaid with lysosomes (red), demonstrating that these H-L-D-Z@PM NPs could enter the cells through the endo-lysosome network. Additionally, after 6-h incubation, more H-L-D-Z@PM NPs were in the cells, and stronger green color was observed in the nuclei, which indicated that DOX was released from the H-L-D-Z@PM NPs progressively and gathered in the nuclei. As we know, the targeting site of DOX is in the nuclei. A high concentration of DOX inside the nuclei indicates the enhanced antitumor effect.

**FIGURE 4:**
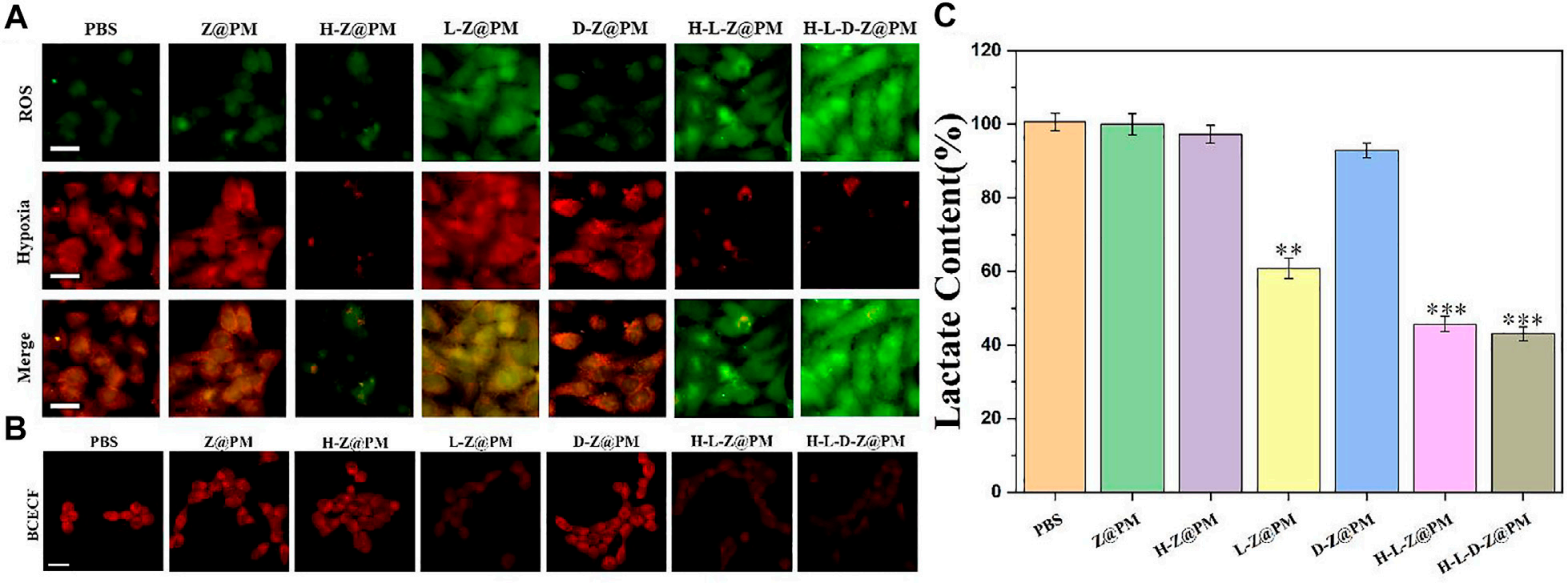
H-L-D-Z@PM-mediated generation of oxidative stress and slightly acidic and hypoxic environment in 4T1 cells. Fluorescence micrographs of 4T1 cells with **(A)** oxidative stress/hypoxia (Scale bar: 20 µm) and CLSM images of 4T1 cells. **(B)** pH (scale bar: 20 µm) detection probes in different treatments. **(C)** Lactate content after incubation with different materials for 24 h **p < 0.01, ***p < 0.001.

To verify the relationship between regulating acidosis and relieving hypoxia by H-L-D-Z@PM NPs, we assessed the cellular hypoxia (Figure 4A) and pH (Figure 4B) by corresponding fluorescent probes. Moreover, the cellular oxidative stress level caused by the depletion of lactate, which is also an important part of promoting tumor cell apoptosis, was also measured (Figure 4A). As reflected in Figure 4A, after co-incubation of these NPs with 4T1 cells for 24 h, almost no ROS was detected in the groups of PBS, Z@PM, H-Z@PM, and D-Z@PM, while different degrees of ROS were seen in the groups of L-Z@PM, H-L-Z@PM, and H-L-D-Z@PM, respectively. We thought that due to the presence of LOX, lactate would be converted to pyruvate and hydrogen peroxide with the participation of oxygen, leading to the high level of ROS inside these cells. Interestingly, severe hypoxia was observed in the L-Z@PM NPs group, while the H-Z@PM, H-L-Z@PM, and H-L-D-Z@PM NPs had less hypoxic signal inside the cancer cells. We thought that the catalyzation of lactate to pyruvate and hydrogen peroxide by LOX would consume oxygen, thus leading to severe hypoxia in the L-Z@PM group. However, in the groups of H-L-Z@PM and H-L-D-Z@PM, even if LOX consumed much oxygen in the catalyzing procedure, the presence of Hb would deliver and release oxygen to alleviate hypoxia effectively.

Furthermore, the acidic degree in the cells was detected by a red fluorescence probe, as depicted in Figure 4B. In the cells treated by NPs without the loading of LOX, red fluorescence is clearly observed (PBS), indicating severe acidosis in the cells. After these cells were incubated with NPs loading the LOX, such as L-Z@PM, H-L-Z@PM, and H-L-D-Z@PM NPs, the red fluorescence intensity was very weak, illustrating that all these NPs can regulate the acidic environment in tumor cells efficiently. We speculated that the decrease in acidosis could be attributed to the depletion of lactate in the presence of oxygen. The degrees of lactate in the cells after different treatments were also evaluated. Without the presence of LOX, the cells showed similar lactate degrees after the treatment of PBS, Z@PM, H-Z@PM, and D-Z@PM NPs, respectively. While in the groups of L-Z@PM, H-L-Z@PM, and H-L-D-Z@PM NPs, the level of lactate greatly descended, which also demonstrated that lactate was converted to pyruvate and hydrogen peroxide. Furthermore, the lactate concentration after the treatment by H-L-Z@PM or H-L-D-Z@PM was less than that of the L-Z@PM group. This phenomenon is reasonable that the depletion process of lactic acid in the presence of LOX would consume oxygen. In the H-L-Z@PM or H-L-D-Z@PM groups, since the hemoglobin was loaded with oxygen, the content of oxygen in tumor cells was increased, thereby promoting the decomposition of lactate and leading to the lower level of lactate. However, in the L-Z@PM group, due to the consumption of oxygen during the decomposition of lactate, the intracellular oxygen content decreased, resulting in a more hypoxic system, which in turn hampered the decomposition of lactate. The severe hypoxia in the L-Z@PM group is also demonstrated in Figure 4A. Collectively, all these data revealed that H-L-D-Z@PM NPs not only effectively depleted the intracellular lactate but also alleviated hypoxia.

**FIGURE 5:**
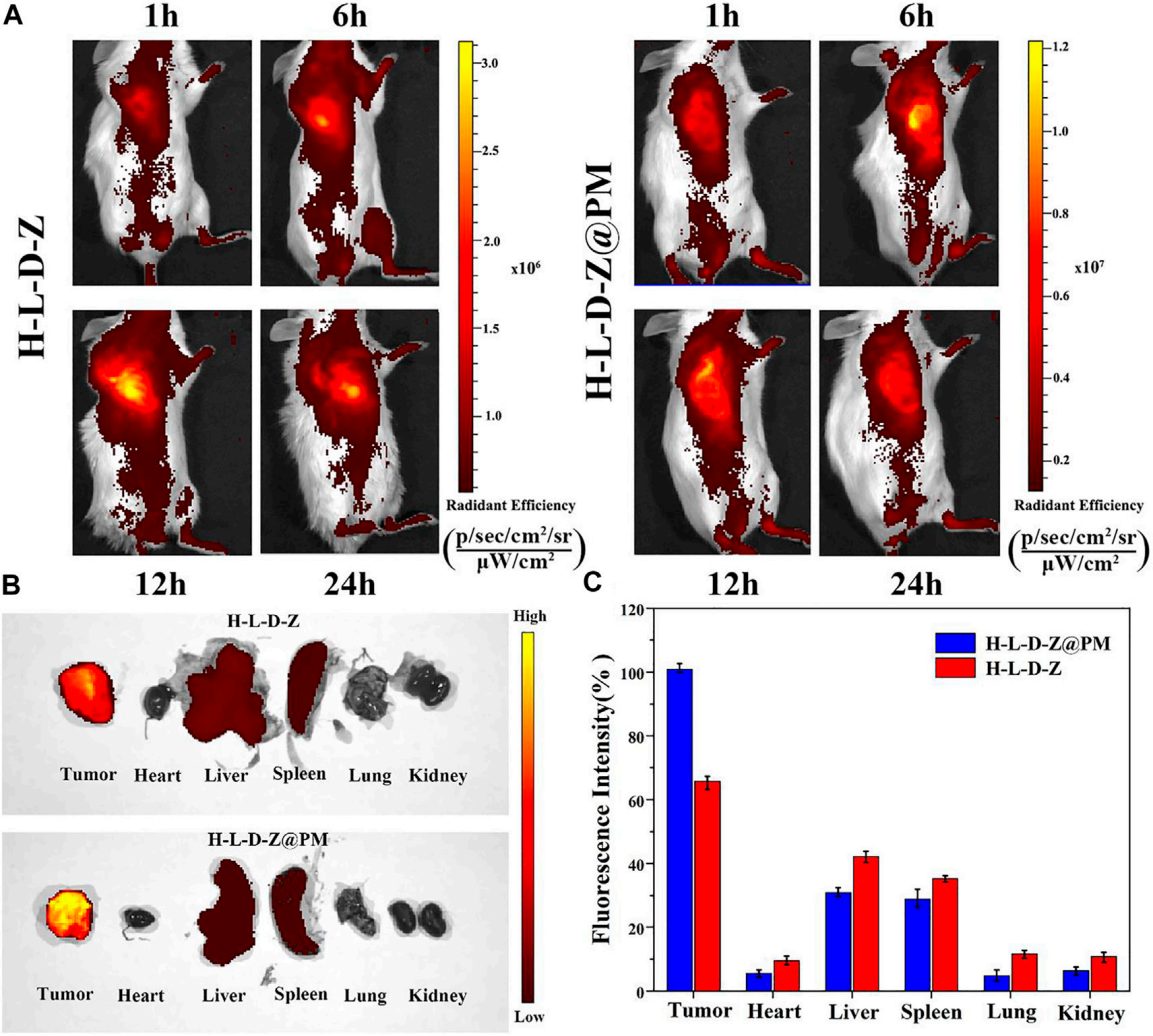
Tumor-targeting capacity of H-L-D-Z NPs and H-L-D-Z@PM NPs. **(A)** *In vivo* fluorescence imaging of mice received an intravenous injection of IR780-labeled H-L-D-Z and H-L-D-Z@PM NPs. **(B)** Fluorescence images of organs harvested at 72 h post-injection. **(C)** Fluorescence intensity statistics of major organs in each treatment group.

The distribution of nanomedicine would greatly affect the *in vivo* antitumor efficiency. A fluorescence imaging experiment was applied to track the *in vivo* bio-distribution of NPs in mice. The H-L-D-Z and H-L-D-Z@PM NPs were labeled with IR780 and intravenously injected into 4T1 tumor-bearing mice, and their luminescence images were taken at different time points (Figure 5A). According to Figure 5A, regardless of coated PM or not, the majority of NPs were enriched in the liver first. As time went by, the NPs accumulated in tumor tissue gradually due to the EPR effect (H-L-D-Z NPs) or the active targeting ability of P-selectin (H-L-D-Z@PM NPs). The largest discrepancy between the H-L-D-Z group and the H-L-D-Z@PM group was that the H-L-D-Z@PM achieved peak fluorescence intensity within 6 h compared to 12 h for the H-L-D-Z groups (Figure 5A). After 72 h of injection, all these mice were killed, and their major organs were collected. Clearly, most of these NPs were in mononuclear phagocyte system-associated organs (such as the spleen and liver). The highest luminescence intensity was observed in tumor tissue (Figure 5B). Of note, after being coated with PM, more H-L-D-Z@PM NPs were distributed in tumor tissue due to the targeting ability of PM. These phenomena demonstrated that PM coating could enhance the tumor accumulation efficiency of H-L-D-Z@PM NPs remarkably.

**FIGURE 6:**
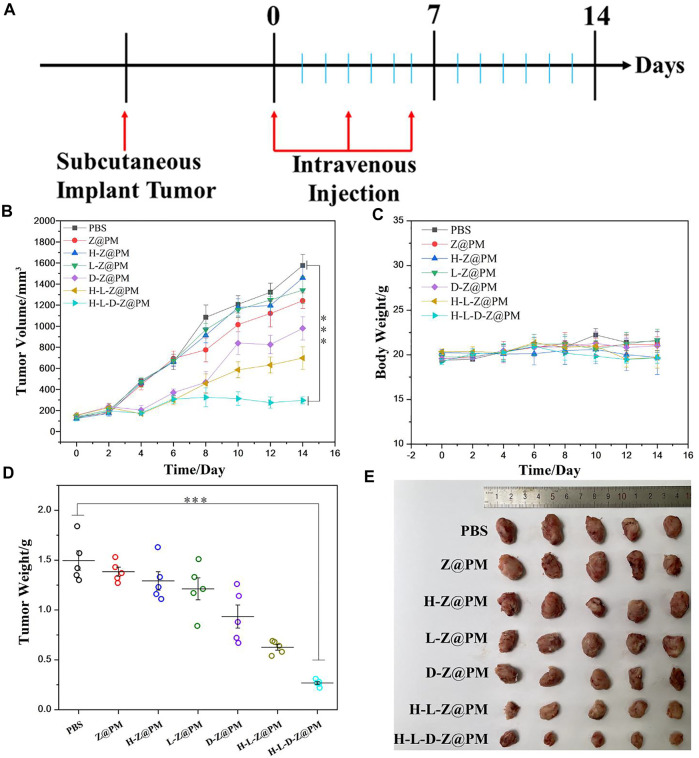
**(A)** Illustration of the experimental design. **(B)** Tumor volume and **(C)** body weight after different treatments. **(D)** Tumor weight after different treatments. **(E)** Photos of tumor dissection. ***p < 0.001.

To comprehensively evaluate the *in vivo* antitumor effect of H-L-D-Z@PM NPs, we proceeded to investigate the tumor inhibition effect of H-L-D-Z@PM NPs using a subcutaneous 4T1 tumor-bearing BALB/c mouse model. These mice were divided into seven groups for different treatments. The treatment regimen is illustrated in Figure 6A. When the tumor volume reached 100 mm^3^, mice received tail vein injections of PBS, Z@PM, H-Z@PM. L-Z@PM, D-Z@PM, H-L-Z@PM, and H-L-D-Z@PM every 3 days for three times, and the tumor volume was recorded every other day (Figure 6B). For the PBS, Z@PM, H-Z@PM, and L-Z@PM treated groups, during the 14-day therapy, tumors continued to grow over time, and at the end of the experiment, they had similar tumor volumes in all groups. The H-L-D-Z@PM NPs appeared to have outstanding tumor-inhibiting capability, and almost no obvious tumor growth was witnessed during the testing period. All the experimental mice did not show remarkable weight loss during the testing period, indicating these samples have no or less acute toxic effect on mice (Figure 6C). Moreover, the mice receiving the treatment of H-L-D-Z@PM NPs showed the lowest tumor weight (Figure 6D) and the smallest tumor size (Figure 6E), also indicating the best antitumor effect of H-L-D-Z@PM NPs.

**FIGURE 7:**
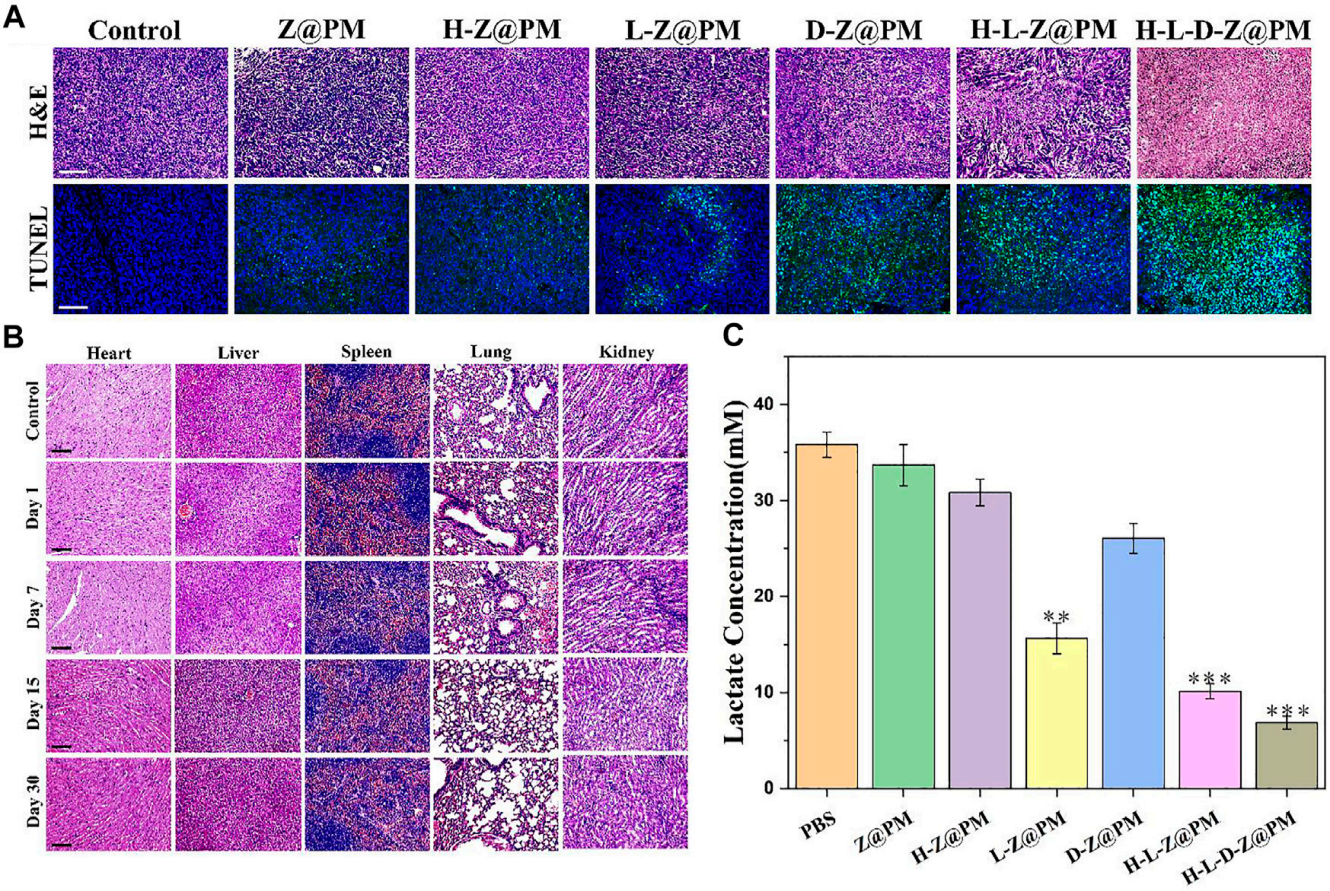
**(A)** Fluorescence microscopy images of H and E- and TUNEL-stained tumor sections after different treatments. Scale bar: 100 µm. **(B)** Long-term toxicity of H-L-D-Z@PM NPs in the heart, liver, spleen, lungs, and kidney at days 1, 7, 15, and 30 using H and E staining. Scale bar: 100 µm. **(C)** Tumor lactate concentration after different treatments. **p < 0.01, ***p < 0.001.

To further evaluate the therapeutic effect and long-term biological toxicity of H-L-D-Z@PM and other NPs, at the end of 14-day therapy, tumor tissues in each group were collected and stained with hematoxylin and eosin (H and E), and the histological changes of tumors were investigated (Figure 7A). Apparently, despite the varying extent of damage that was found, the most grievous damage in tumor tissue was witnessed in the H-L-D-Z@PM NPs group while no obvious impairment was observed in the PBS group. The appraisal of terminal deoxynucleotidyl transferase (TdT)-mediated dUTP nick-end labeling (TUNEL) assay further demonstrated the aforementioned observation, with massive apoptotic cells in the whole tumor tissue. As shown in Figure 7B, compared to normal mice, there is no conspicuous damage in the major organs of the mice after the final treatment, which implies the good biocompatibility of H-L-D-Z@PM NPs. We employed golden standard pimonidazole-staining in addition to immuno-staining of HIF-1α to visualize hypoxic cells that differ in hypoxia conditions. As shown in Supplementary Figure S3 in our revised manuscript, mice that received the treatment of H-L-D-Z@PM exhibited pronounced alleviation in hypoxia conditions compared to mice treated with PBS (control). The removal of hemoglobin (H for short in H-L-D-Z@PM) substantially weakens the capacity of the nano-platform to mitigate hypoxia, attributing the hypoxia alleviation to the oxygen cargo released from hemoglobin. Finally, we assessed the lactate concentration in tumor tissue after various treatments (Figure 7C). It is evident that there is the lowest content of lactate in the tumor tissue of the H-L-D-Z@PM NPs treated group, which could be attributed to the synergism of LOX and enough oxygen in this group. Therefore, all these findings verified that H-L-D-Z@PM NPs could not only obliterate the cancer cells without obvious toxic effects but also, more importantly, remodel the tumor acid environment to repress the growth of cancer cells.

## Conclusion

In summary, we developed a multi-functional nano-platform of H-L-D-Z@PM NPs for the inhibition of tumor growth. In this system, PM not only enhanced the tumor-targeting ability of H-L-D-Z@PM NPs but also improved the biocompatibility of nano-platform. LOX was used for depleting lactate to regulate acidosis. Hb is intended for delivering oxygen to relieve hypoxia in tumor tissue and boost the catalytic activity of LOX. DOX is applied for chemotherapy to strengthen the inhibition of tumor growth. ZIF8 is designed to meet the needs of encapsulating Hb, LOX, and DOX. Meanwhile, it would efficiently dissociate and release the payload inside tumor cells. Both *in vitro* and *in vivo* experiments have verified that the H-L-D-Z@PM can deplete lactate and relieve hypoxia efficiently. We hope that our contrivance could provide a promising method for cancer therapy.

## Data Availability

The raw data supporting the conclusions of this article will be made available by the authors, without undue reservation.

## References

[B1] Binnewies M. Roberts E. W. Kersten K. Chan V. Fearon D. F. Merad M. (2018). Understanding the Tumor Immune Microenvironment (TIME) for Effective Therapy. Nat. Med. 24, 541–550. 10.1038/s41591-018-0014-x 29686425 PMC5998822

[B2] Ferrari M. (2005). Cancer Nanotechnology: Opportunities and Challenges. Nat. Rev. Cancer 5, 161–171. 10.1038/nrc1566 15738981

[B3] Gao F. Tang Y. Liu W. L. Zou M. Z. Huang C. Liu C. J. (2019). Intra/Extracellular Lactic Acid Exhaustion for Synergistic Metabolic Therapy and Immunotherapy of Tumors. Adv. Mater. 31, e1904639. 10.1002/adma.201904639 31692128

[B4] Harris A. L. (2002). Hypoxia - A Key Regulatory Factor in Tumour Growth. Nat. Rev. Cancer 2, 38–47. 10.1038/nrc704 11902584

[B5] Hu C.-M. J. Fang R. H. Wang K.-C. Luk B. T. Thamphiwatana S. Dehaini D. 2015. Nanoparticle Biointerfacing by Platelet Membrane Cloaking. Nature *,* 526, 118, 121. 10.1038/nature15373 26374997 PMC4871317

[B6] Huo D. Zhu J. Chen G. Chen Q. Zhang C. Luo X. (2019). Eradication of Unresectable Liver Metastasis through Induction of Tumour Specific Energy Depletion. Nat. Commun. 10, 3051. 10.1038/s41467-019-11082-3 31296864 PMC6624273

[B7] Jiang W. Han X. Zhang T. Xie D. Zhang H. Hu Y. (2020). An Oxygen Self-Evolving, Multistage Delivery System for Deeply Located Hypoxic Tumor Treatment. Adv. Healthc. Mater. 9, e1901303. 10.1002/adhm.201901303 31823515

[B8] Jiang W. Luo X. Wei L. Yuan S. Cai J. Jiang X. (2021). The Sustainability of Energy Conversion Inhibition for Tumor Ferroptosis Therapy and Chemotherapy. Small 17, e2102695. 10.1002/smll.202102695 34350694

[B9] Jin Z. Y. Zhao Q. Y. Yuan S. M. Jiang W. Hu Y. (2021). Strategies of Alleviating Tumor Hypoxia and Enhancing Tumor Therapeutic Effect by Macromolecular Nanomaterials. Macromolecular Biosci. 21, e2100092. 10.1002/mabi.202100092 34008312

[B10] Li B. Chu T. Wei J. Zhang Y. Qi F. Lu Z. (2021). Platelet-Membrane-Coated Nanoparticles Enable Vascular Disrupting Agent Combining Anti-angiogenic Drug for Improved Tumor Vessel Impairment. Nano Lett. 21, 2588–2595. 10.1021/acs.nanolett.1c00168 33650872

[B11] Liu G. Zhao X. Zhang Y. Xu J. Xu J. Li Y. (2019a). Engineering Biomimetic Platesomes for pH-Responsive Drug Delivery and Enhanced Antitumor Activity. Adv. Mater. 31, e1900795. 10.1002/adma.201900795 31222856

[B12] Liu J. J. Jin Y. J. Song Z. Xu L. H. Yang Y. Zhao X. (2021). Boosting Tumor Treatment by Dredging the Hurdles of Chemodynamic Therapy Synergistic Ion Therapy. Chem. Eng. J. 411, 128440. 10.1016/j.cej.2021.128440

[B13] Liu S. Luo X. Liu S. Xu P. Wang J. Hu Y. (2019b). Acetazolamide-Loaded pH-Responsive Nanoparticles Alleviating Tumor Acidosis to Enhance Chemotherapy Effects. Macromol Biosci. 19, e1800366. 10.1002/mabi.201800366 30511819

[B14] Otsuka Y. Kiyohara C. Kashiwado Y. Sawabe T. Nagano S. Kimoto Y. (2018). Effects of Tumor Necrosis Factor Inhibitors and Tocilizumab on the Glycosylated Hemoglobin Levels in Patients with Rheumatoid Arthritis; an Observational Study. Plos One 13, e0196368. 10.1371/journal.pone.0196368 29694426 PMC5918963

[B15] Pillai S. R. Damaghi M. Marunaka Y. Spugnini E. P. Fais S. Gillies R. J. (2019). Causes, Consequences, and Therapy of Tumors Acidosis. Cancer Metastasis Rev. 38, 205–222. 10.1007/s10555-019-09792-7 30911978 PMC6625890

[B16] Rohani N. Hao L. Alexis M. S. Joughin B. A. Krismer K. Moufarrej M. N. (2019). Acidification of Tumor at Stromal Boundaries Drives Transcriptome Alterations Associated with Aggressive Phenotypes. Cancer Res. 79, 1952–1966. 10.1158/0008-5472.can-18-1604 30755444 PMC6467770

[B17] Saha S. Xiong X. Chakraborty P. K. Shameer K. Arvizo R. R. Kudgus R. A. (2016). Gold Nanoparticle Reprograms Pancreatic Tumor Microenvironment and Inhibits Tumor Growth. Acs Nano 10, 10636–10651. 10.1021/acsnano.6b02231 27758098 PMC6939886

[B18] Semenza G. L. (2003). Targeting HIF-1 for Cancer Therapy. Nat. Rev. Cancer 3, 721–732. 10.1038/nrc1187 13130303

[B19] Tang J. Meka A. K. Theivendran S. Wang Y. Yang Y. Song H. (2020). Openwork@Dendritic Mesoporous Silica Nanoparticles for Lactate Depletion and Tumor Microenvironment Regulation. Angew. Chem. Int. Ed. 59, 22054–22062. 10.1002/anie.202001469 32705778

[B20] Tian Y. Li S. Song J. Ji T. Zhu M. Anderson G. J. (2014). A Doxorubicin Delivery Platform Using Engineered Natural Membrane Vesicle Exosomes for Targeted Tumor Therapy. Biomaterials 35, 2383–2390. 10.1016/j.biomaterials.2013.11.083 24345736

[B21] Voskuil F. J. Steinkamp P. J. Zhao T. Van Der Vegt B. Koller M. Doff J. J. (2020). Exploiting Metabolic Acidosis in Solid Cancers Using a Tumor-Agnostic pH-Activatable Nanoprobe for Fluorescence-Guided Surgery. Nat. Commun. 11, 3257. 10.1038/s41467-020-16814-4 32591522 PMC7320194

[B22] Wang W. Zhang L. Deng Q. Liu Z. Ren J. Qu X. (2022). Yeast@MOF Bioreactor as a Tumor Metabolic Symbiosis Disruptor for the Potent Inhibition of Metabolically Heterogeneous Tumors. Nano Today 42, 101331. 10.1016/j.nantod.2021.101331

[B23] Warburg O. Wind F. Negelein E. (1927). The Metabolism of Tumors in the Body. J. Gen. Physiol. 8, 519–530. 10.1085/jgp.8.6.519 19872213 PMC2140820

[B24] Welter M. Fredrich T. Rinneberg H. Rieger H. (2016). Computational Model for Tumor Oxygenation Applied to Clinical Data on Breast Tumor Hemoglobin Concentrations Suggests Vascular Dilatation and Compression. Plos One 11, e0161267. 10.1371/journal.pone.0161267 27547939 PMC4993476

[B25] Xia D. Hang D. Li Y. Jiang W. Zhu J. Ding Y. (2020). Au-Hemoglobin Loaded Platelet Alleviating Tumor Hypoxia and Enhancing the Radiotherapy Effect with Low-Dose X-ray. Acs Nano 14, 15654–15668. 10.1021/acsnano.0c06541 33108152

[B26] Xia D. L. Xu P. P. Luo X. Y. Zhu J. F. Gu H. Y. Huo D. (2019). Overcoming Hypoxia by Multistage NP Delivery System to Inhibit Mitochondrial Respiration for Photodynamic Therapy. Adv. Funct. Mater. 29, 1807294. 10.1002/adfm.201807294

[B27] Yang J. Li W. Luo L. Jiang M. Zhu C. Qin B. (2018). Hypoxic Tumor Therapy by Hemoglobin-Mediated Drug Delivery and Reversal of Hypoxia-Induced Chemoresistance. Biomaterials 182, 145–156. 10.1016/j.biomaterials.2018.08.004 30121013

[B28] Yuan M. Liang S. Zhou Y. Xiao X. Liu B. Yang C. (2021). A Robust Oxygen-Carrying Hemoglobin-Based Natural Sonosensitizer for Sonodynamic Cancer Therapy. Nano Lett. 21, 6042–6050. 10.1021/acs.nanolett.1c01220 34254814

[B29] Zhang C. Xia D. L. Liu J. H. Huo D. Jiang X. Q. Hu Y. (2020). Bypassing the Immunosuppression of Myeloid-Derived Suppressor Cells by Reversing Tumor Hypoxia Using a Platelet-Inspired Platform. Adv. Funct. Mater. 30, 2000189. 10.1002/adfm.202000189

[B30] Zhang L. Xia J. Zhao Q. Liu L. Zhang Z. (2010). Functional Graphene Oxide as a Nanocarrier for Controlled Loading and Targeted Delivery of Mixed Anticancer Drugs. Small 6, 537–544. 10.1002/smll.200901680 20033930

[B31] Zhang M. Ye J.-J. Xia Y. Wang Z.-Y. Li C.-X. Wang X.-S. (2019). Platelet-Mimicking Biotaxis Targeting Vasculature-Disrupted Tumors for Cascade Amplification of Hypoxia-Sensitive Therapy. Acs Nano 13, 14230–14240. 10.1021/acsnano.9b07330 31714733

[B32] Zhang X. K. Yang S. B. Wang Q. Ye W. M. Liu S. L. Wang X. (2021). Tailored Theranostic NPs Cause Efficient Ferroptosis in Head and Neck Squamous Cell Carcinoma through a Reactive Oxygen Species "butterfly Effect. Chem. Eng. J. 423, 130083. 10.1016/j.cej.2021.130083

[B33] Zheng C. Wang Y. Phua S. Z. F. Lim W. Q. Zhao Y. (2017). ZnO-DOX@ZIF-8 Core-Shell Nanoparticles for pH-Responsive Drug Delivery. ACS Biomater. Sci. Eng. 3, 2223–2229. 10.1021/acsbiomaterials.7b00435 33445281

[B34] Zheng R. S. Sun K. X. Zhang S. W. Zeng H. M. Zou X. N. Chen R. (2019). Report of Cancer Epidemiology in China, 2015. Zhonghua Zhong Liu Za Zhi 41, 19–28. 10.3760/cma.j.issn.0253-3766.2019.01.005 30678413

[B35] Zhu S. Gu Z. J. Zhao Y. L. (2018). Harnessing Tumor Microenvironment for NP-Mediated Radiotherapy. Adv. Ther. 1, 1800050. 10.1002/adtp.201800050

