# Supplementary material for: Regulating Acidosis and Relieving Hypoxia by Platelet Membrane-Coated Nanoparticle for Enhancing Tumor Chemotherapy
Source: Front Bioeng Biotechnol. 2022 May 12;10:885105. doi: 10.3389/fbioe.2022.885105 (PMC9135319; doi:10.3389/fbioe.2022.885105)
Supplement: Supplementary file 1 [file DataSheet1.docx]

Supplementary Material

Regulating Acidosis and Relieving Hypoxia by Platelet Membrane Coated Nanoparticle for Enhancing Tumor Chemotherapy

Xingyu Luo^1^, Jian Cao^1^, Jianming Yu^1^, Dongqing Dai^2^, Wei Jiang^1*^, Yahui Feng^2^^*^, Yong Hu^1*^

^1^College of Engineering and Applied Sciences, MOE Key Laboratory of High Performance Polymer Materials & Technology, Nanjing University, Nanjing, China, 210033

^2^Nanjing Customs District Industrial Products Inspection Center, 39 Chuangzhi Road, Nanjing, China. 210019

*** Correspondence:** [hvyong@nju.edu.cn](mailto:hvyong@nju.edu.cn); [eggshell618@126.com](mailto:eggshell618@126.com); 604095024@qq.com

Keywords: Nanoparticles, Chemotherapy, Lactate Oxidase, Platelet Membrane, Tumor Microenvironment.


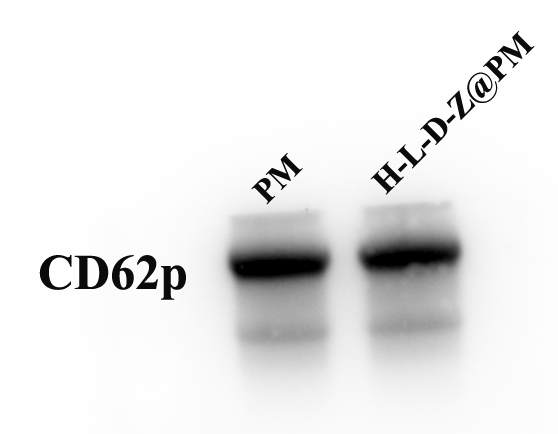


**Supplementary Figure 1.** Western blot analysis of the p-selectin proteins in platelet membranes, and H-L-D-Z@PM

**

**

**Supplementary Figure 2** Standard curve line of DOX


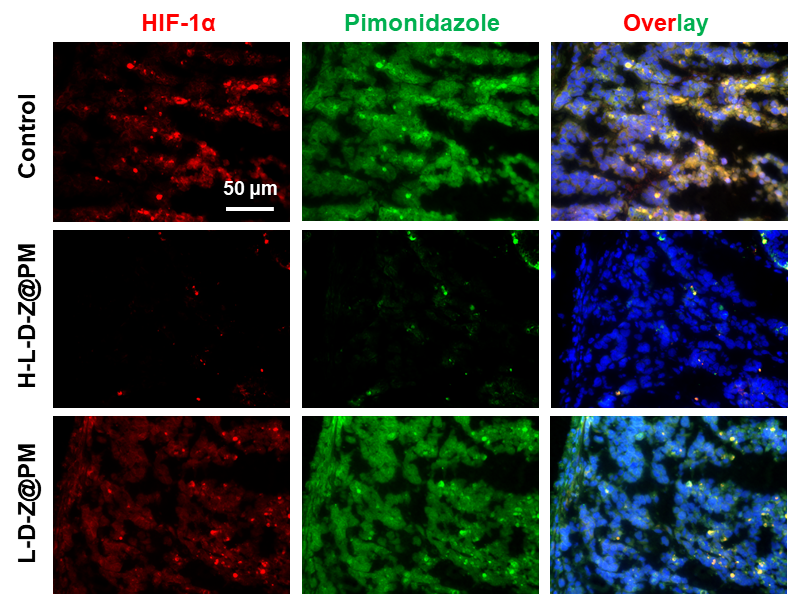


**Supplementary Figure 3** The fluorescence microscopy images of HIF-1α and Pimonidazole-stained tumor sections after different treatment
